# Sclerosing encapsulating peritonitis in a dog with pancreatic ductal adenocarcinoma

**DOI:** 10.1186/s12917-022-03485-0

**Published:** 2022-11-02

**Authors:** Yusuke Tsukada, Young Tae Park, Ikki Mitsui, Masahiro Murakami, Atsushi Tsukamoto

**Affiliations:** 1 Matsuyama Hojo Veterinary Clinic, Hojo, Matsuyama, Ehime 799-2431 Japan; 2 Ve. C. Jiyugaoka Animal Medical Center, 3-24-9, Yakumo, Meguro-ku, Tokyo, Japan; 3 Laboratory of Veterinary Anatomy, Faculty of Veterinary Medicine, Okayama University of Science, 1-3 Ikoi-no-oka, Imabari-shi, Ehime 794-8555 Japan; 4 Department of Veterinary Clinical Sciences, College of Veterinary Medicine, Purdue University, 625 Harrison Street, West Lafayette, IN 47907 USA; 5 Laboratory of Laboratory Animal Science, Azabu University, School of Veterinary Medicine, 1-17-71 Fuchinobe, Chuou-ku, Sagamihara, Kanagawa 252-5201 Japan

**Keywords:** Sclerosing encapsulating peritonitis, Pancreatic ductal adenocarcinoma, Laparoscopy, Dogs

## Abstract

**Background:**

Sclerosing encapsulating peritonitis (SEP) is a rare clinical syndrome characterised by fibrosis and thickening of the peritoneum with massive adhesions of the abdominal organs. In humans, abdominal tumours, such as pancreatic adenocarcinoma, can be underlying diseases of SEP. This report describes a case of SEP in a dog with pancreatic ductal adenocarcinoma.

**Case presentation:**

An 11-year-old male neutered French Bulldog presented with chronic vomiting. Ultrasonography revealed a mass in the centre of the abdomen. A small amount of ascites, interpreted as modified transudate, was present in the abdominal cavity. Computed tomography (CT) revealed peritoneal effusion with a thickened peritonium. Laparoscopy revealed a large nodular lesion occupying the central portion of the abdomen, continuous with the falciform ligament. Histological examination of the biopsy specimens of the mass, abdominal wall, and gastric peritoneum revealed marked fibroplasia with mild lymphoplasmacytic infiltrates. Based on these results, a tentative diagnosis of early stage sclerosing encapsulating peritonitis (SEP) was made. Prednisolone and tamoxifen were administered with the expectation of ameliorating SEP, however, the dog died 61 days post diagnosis. At autopsy, the intestinal loop and mesentery were encased in the fibrous membrane, which is a typical finding in SEP. Histopathology and immunohistochemistry of the samples obtained at autopsy supported the diagnosis of pancreatic ductal adenocarcinoma with peritoneal dissemination and distant metastasis with desmoplasia. The unexpectedly hardened skin, where previously laparoscopic ports were inserted, histologically contained the same carcinoma cells with desmoplasia.

**Conclusions:**

To the best of our knowledge, this is the first report of canine SEP with pancreatic ductal adenocarcinoma that also caused metastasis to port insertion sites as well as distant organs.

## Background

Sclerosing encapsulating peritonitis (SEP) is a chronic form of peritonitis characterised by fibrosis and thickening of the visceral and parietal peritoneum with massive adhesions of the abdominal organs [1]. Peritoneal inflammation and intestinal adhesion cause clinical symptoms, such as abdominal pain, anorexia, and vomiting. In humans, the clinical features of SEP are well described, and the major underlying causes are peritoneal dialysis, abdominal surgery, drug administration, and abdominal tumours [2]. In contrast, it is an extremely rare condition in dogs, and the aetiology of SEP is not fully understood, but is thought to be multifactorial [3]. Most cases of canine SEP are diagnosed at the time of exploratory laparotomy or autopsy [1, 3–5]. Treatment protocols for SEP in dogs have not yet been established; therefore, SEP has a poor prognosis in most cases.

Pancreatic carcinoma arising from exocrine acinar cells or duct epithelial cells is rare in veterinary medicine [6]. Early diagnosis of this tumour in dogs is difficult, and a previous study reported that metastasis was observed in 78% of cases at the time of diagnosis [7]. Although pancreatic ductal carcinomas are a minority among canine pancreatic carcinomas, this type of carcinoma is consistently associated with abdominal or distant metastasis [6]. In humans, pancreatic ductal carcinomas cause prominent fibrosis of the pancreas, which may lead to SEP [8, 9]. This report describes a case of SEP associated with pancreatic ductal adenocarcinoma in a dog.

## Case presentation

**Fig. 1:**
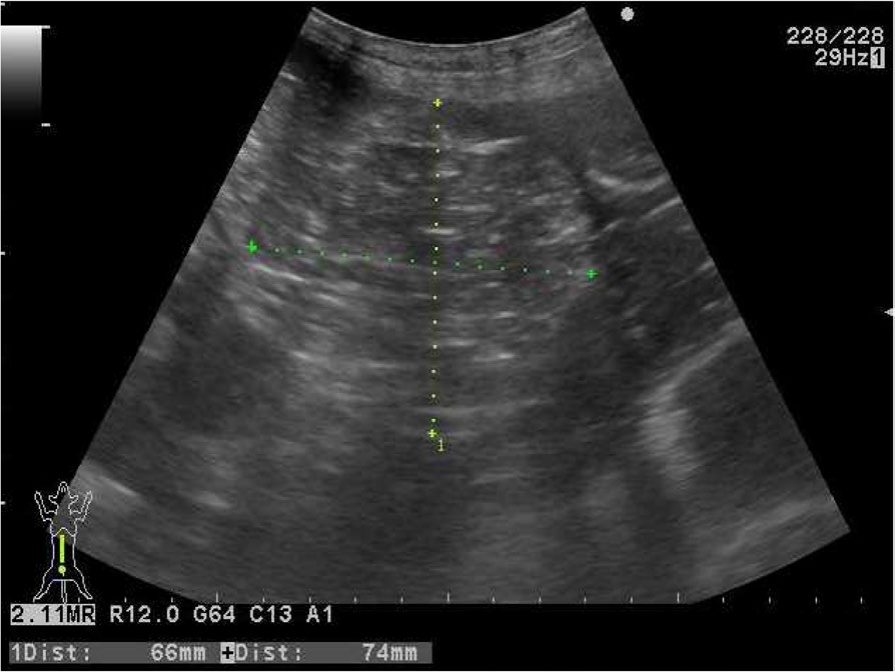
Ultrasonographic image of abdominal mass located in the centre of the abdomen

An 11-year-old male neutered French Bulldog with a history of chronic vomiting was referred to our hospital. A physical examination revealed abdominal pain and swelling. Blood analysis showed mild elevation of creatinine kinase (229 U/L; range 10–200 U/L). Abdominal radiography revealed a caudal displacement of the gastrointestinal tract by mixed fat and soft tissue opacity in the cranial to mid abdomen. No radiographic evidence of intestinal obstruction was noted. Ultrasonography revealed a mass with a mixed-echo pattern between the stomach and the intestinal tract, with a small amount of ascites (Fig. 1). Corrugation of the jejunum and ileum was observed. Cytological examination of the ascites revealed a modified transudate, mainly composed of neutrophils and macrophages (specific gravity, 1.020; total protein: 2.2 mg/dL). The bacterial culture of ascites was negative. Following these tests, CT and laparoscopy were performed under general anaesthesia.

**Fig. 2:**
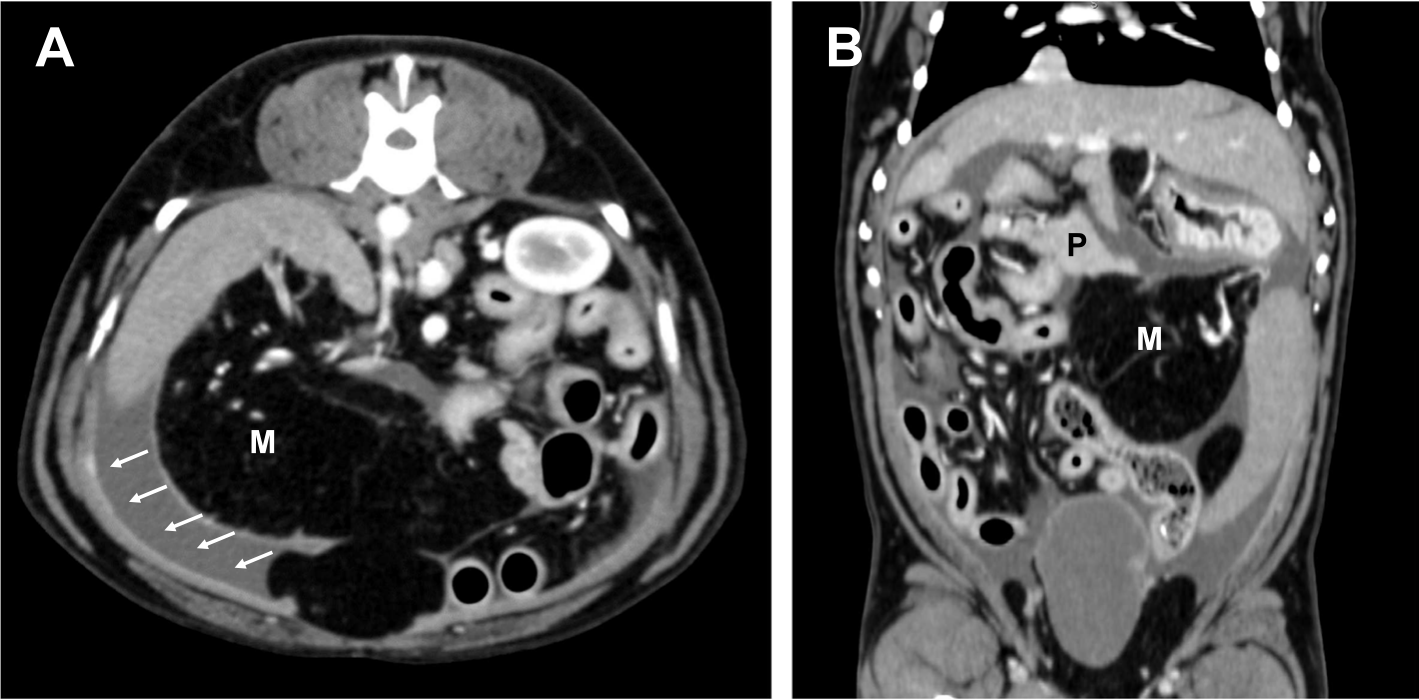
Images of abdominal computed tomography (CT) scan. Transverse (**A**) and dorsal (**B**) CT images after injection of contrast medium. M: mass lesion; P: pancreas. The arrow indicates the peritoneal effusion with a thickened peritoneal line

Pre- and post-intravenous contrast CT were reviewed by a ACVR board-certified radiologist (M.M.) which revealed a moderate amount of non-enhancing fluid attenuation in the peritoneal cavity surrounding and displacing the mesentery and omental fat from the abdominal wall (Fig. 2). In addition, there was mild thickening of the soft tissue attenuating and mildly contrast-enhancing rims of the mesentery, omental fat, and fat along the linea alba adjacent to the peritoneal effusion. The left limb of the pancreas was enlarged with an irregularly rounded margination and homogeneous parenchyma, which iso-attenuated to the rest of the pancreas.

**Fig. 3:**
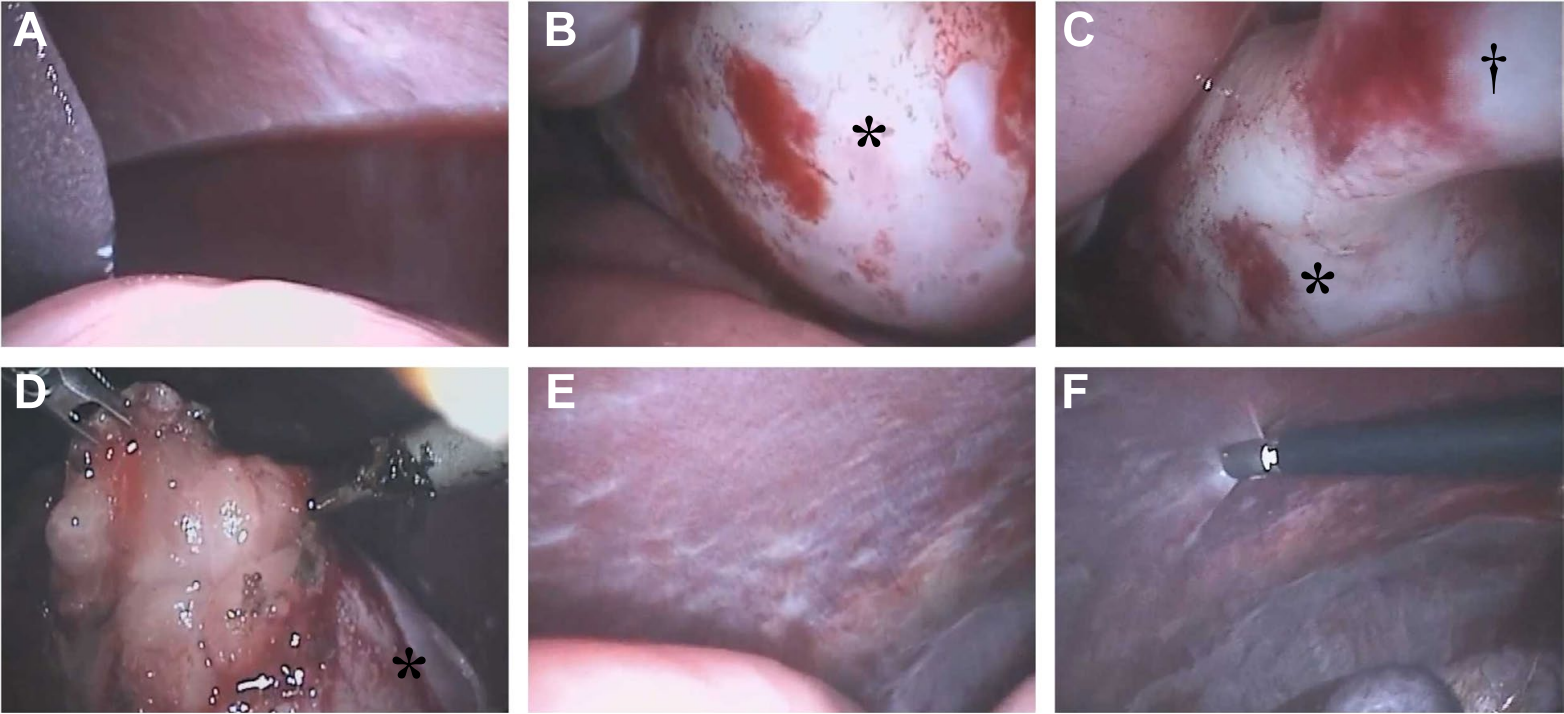
Laparoscopic views of the abdomen. Haemorrhagic ascites was observed in the caudal side of the spleen (**A**). Mass lesion was presented in the centre of the abdomen continuous to the hepatic falciform ligament (**B**, **C**). After excising the surface of the mass lesion, fatty or fat-like tissue emerged from the inside (**D**). The parietal peritoneal surface was diffusely white and rough (E). Samples were taken from parietal peritoneal surface (F). *indicates the mass lesion. †indicates the hepatic falciform ligament

**Fig. 4:**
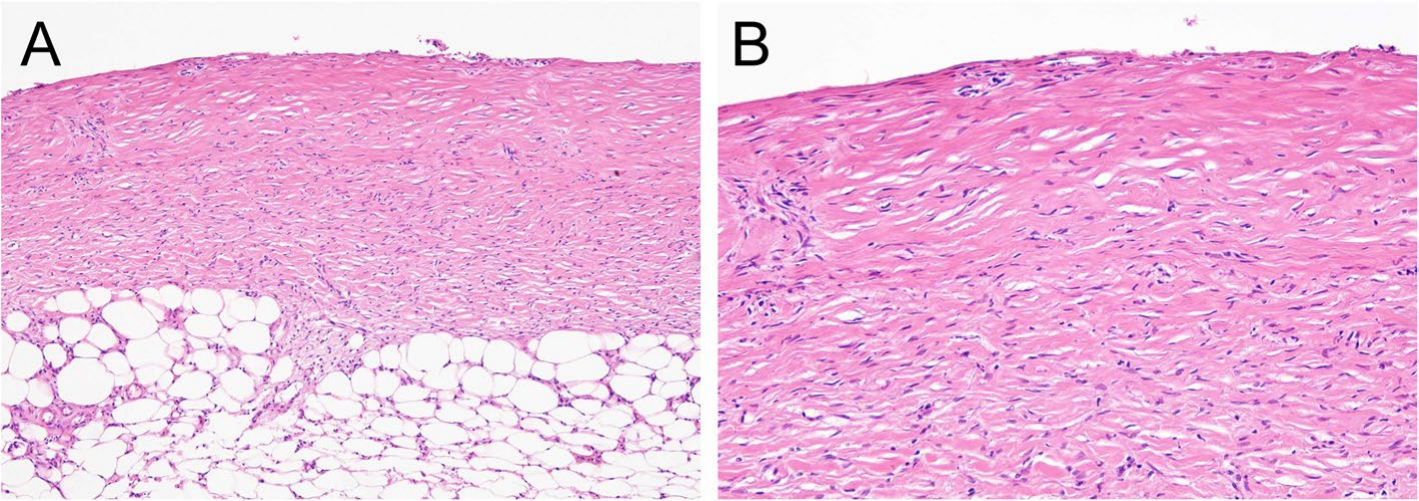
Histopathological findings of biopsy specimen. Samples were taken from the surface of the mass lesion through laparoscopy and stained with haematoxylin-eosin (HE). Photomicrograph shows a thick layer of fibrous connective tissue covering mature adipose tissue. The mesothelial lining is lacking. A: low power field; B: high power field

Laparoscopy revealed a large nodular lesion occupying the central portion of the abdomen, with sanguineous abdominal fluid. The nodular lesion was continuous with the falciform ligament. The parietal peritoneal surface was diffusely white and rough (Fig. 3). Under laparoscopy, biopsies were obtained from the surface of the tumour, the abdominal wall around the tumour, and the serous membrane of the stomach. Histological examination of all samples showed proliferation of fibroconnective tissue with a chronic inflammatory reaction (Fig. 4). The fibroblasts in these tissues showed no atypia. Based on these findings, the dog was presumptively diagnosed with early phase SEP.

Oral prednisolone (1 mg/kg, q24 h) was initiated to reduce fibroplasia and inflammation. Tramadol (5 mg/kg, iv, q12h) was used for pain management. Ascites was drained once per week (1000–1500 mL at each occasion). Two weeks after the initiation of these treatments, the dog exhibited decreased activity, anorexia, and weight loss. Physical examination revealed a marked swelling at the previous laparoscopic port insertion sites. The prednisolone dose was increased to 1.5 mg/kg q24 h. A week later, the dog showed a slight improvement in activity and appetite. Tamoxifen (1 mg/kg, PO, SID) was added to ameliorate peritoneal fibrosis in accordance with a previous report on canine SEP [4]. Despite these treatments, the dog’s condition progressively deteriorated, and the dog died 61 days post-diagnosis.

**Fig. 5:**
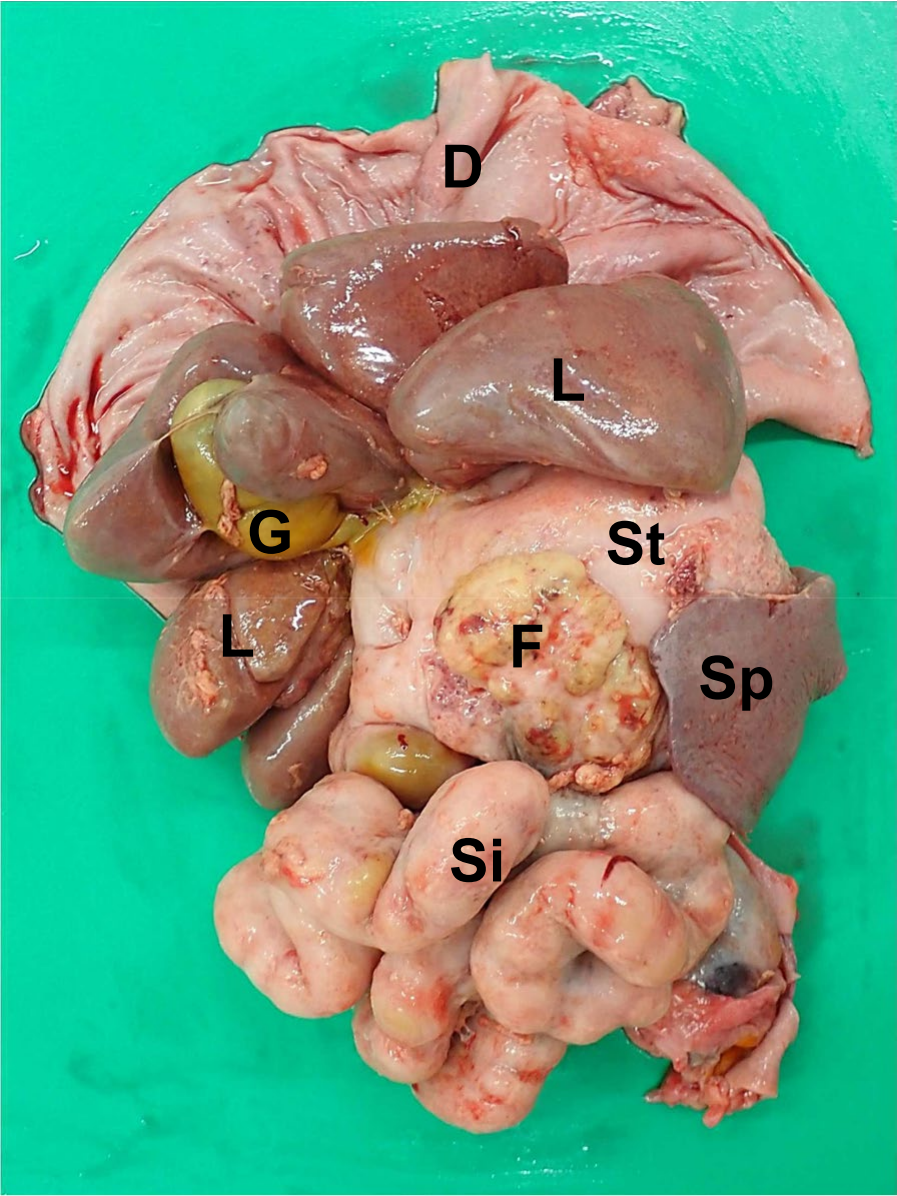
Gross findings of abdominal organs at autopsy. The visceral peritoneum is diffusely thickened by fibrosis. Abdominal organs adhere to each other. Neoplastic nodule/mass is not grossly evident. D: diaphragm; F: fatty appendage of the falciform ligament. G: gallbladder; L: liver; Si: small intestine; Sp: spleen; St: stomach

**Fig. 6:**
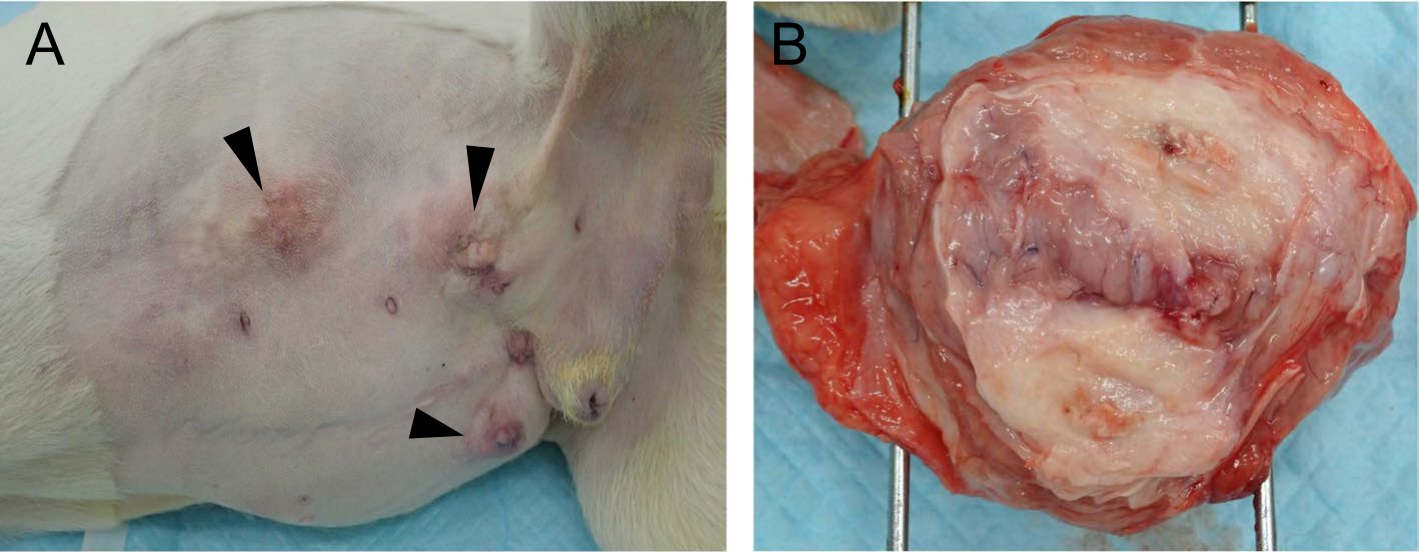
Gross findings of three laparoscopy ports at autopsy. **A**: Ports (arrow heads) were firm and nodular without exuding materials. **B**: Cut section of the hardened laparoscopy port. Subcutaneous fat was replaced by solid fibrous tissue with a gritty to hard central area

A full autopsy of the refrigerated carcass was performed 52 h post-mortem by an ACVP/JCVP board-certified anatomic pathologist (I.M.) with the consent of the owner of the dog. Turbid, 800 ml, dark brown ascites was present. Mildly turbid, orange thoracic fluid (180 mL) was observed. The parietal and visceral peritoneum was diffusely white to pale tan and severely thickened by up to 2 mm. This markedly thickened peritoneum encased the liver and small intestinal loop, resulting in immobilisation of these organs (Fig. 5). During autopsy, close evaluation of the pancreas was restricted by peritoneal fibrosis. The subcutaneous tissue at three previous laparoscopic ports insertion sites (right and left flank and left to the prepuce) was replaced with solid white tissue (Fig. 6A, B). The noncollapsing lung lobes were mottled orange, dark red, and rubbery on palpation. There was a cloudy red serous fluid discharge from the cut surfaces of the lung lobes. A small amount of similar fluid was present in the trachea.

**Fig. 7:**
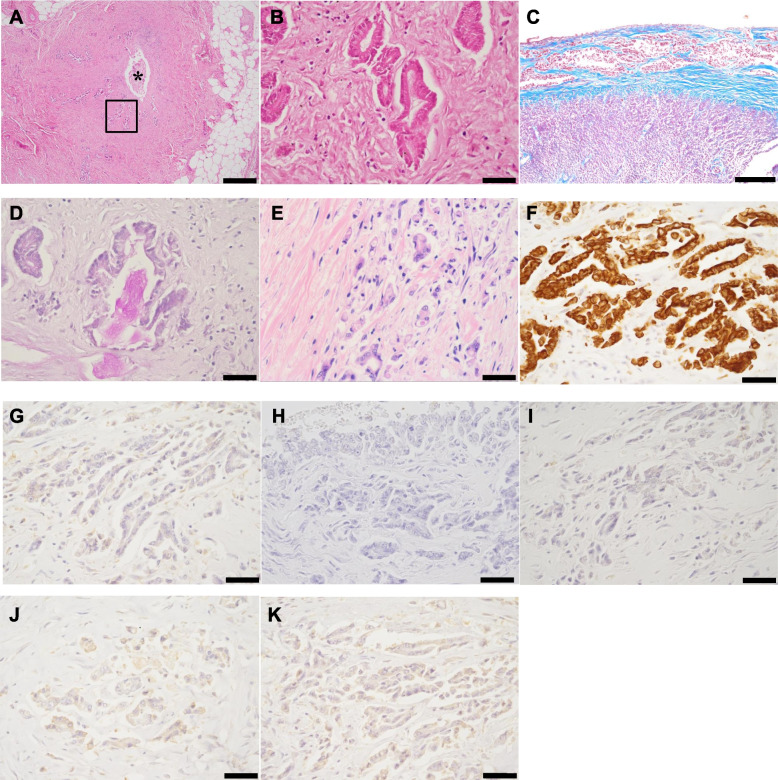
Histopathological and immunohistochemical findings of pancreatic ductal adenocarcinoma of a dog. **A**: Tumour cells proliferate around the pancreatic duct (asterisk). HE stain. (Bar = 400 μm). **B**: A magnified view of the rectangle in Fig. 6A. Tall columnar neoplastic epithelial cells proliferate forming irregular-shape ducts (HE stain; bar = 40 μm). **C**: Desmoplastic fibroplasia around tumour cells in the subserosa of the small intestine (Masson Trichrome stain; Bar = 200 μm). **D**: Mucus secreted by tumour cells is periodic acid-Schiff (PAS) reactive (PAS stain; Bar = 40 μm). **E**: Tumour cells in the laparoscopy port resemble those in other sites (HE stain; bar = 40 μm). **F**: Tumour cells in the subserosa are reactive to anti-pan-cytokeratin antibody. Immunohistochemistry (IHC; bar = 40 μm). **G**: Tumour cells in the subserosa are negative to anti-vimentin antibody (IHC; bar = 40 μm). **H**: Tumour cells in the subserosa are negative to anti-CEA antibody (IHC; bar = 40 μm). **I**: Tumour cells in the subserosa are negative to anti-amylase antibody (IHC; bar = 40 μm). **J**: Tumour cells in the subserosa are negative to anti-chromogranin-A antibody (IHC; bar = 40 μm). **K**. Tumour cells in the subserosa are negative to anti- Wilms tumour 1 (WT1) antibody; (IHC; bar = 40 μm)

Histopathologically, the intestinal loop was diffusely covered by up to 2-mm-thick dense fibrous connective tissue (desmoplasia), with scattered neoplastic foci (Fig. 7A-C). The neoplasm was characterised by haphazardly arranged ducts and tubules of atypical cuboidal to columnar epithelial cells, with secretion of periodic acid-Schiff (PAS)-positive mucus (Fig. 7D). Mitosis was 1 × 2.37 mm^2^ (10 HPFs). Similar neoplastic epithelial cells proliferated around the pancreatic ducts, with desmoplasia. Greater atypia and pleomorphism of the tumour cells were observed among neoplastic cells in the metastatic lesions, such as the lung, right periadrenal tissue, and subcutis of the previous laparoscopy ports insertion sites, than in the pancreatic and peritoneal regions (Fig. 7E). Tumour-associated desmoplasia was a consistent feature of this dog’s neoplasm. No inflammatory infiltrates were observed within or around the neoplasms. The differential diagnosis of this dog’s neoplasm included pancreatic ductal adenocarcinoma, pancreatic acinar cell carcinoma, pancreatic neuroendocrine tumour, and malignant mesothelioma. Advanced characterisation of the tumour cells was performed by a panel of immunohistochemistry (IHC) tests. Information on antibodies and immunohistochemistry results are summarised in Tables 1 and 2, respectively. Briefly, the tumour cells within the pancreas and peritoneum were reactive to CK, while they did not show reactivity to vimentin, CEA, WT1, CGA, or amylase (Table 2 and Fig. 7F-K). Based on these results, the dog was diagnosed with SEP secondary to pancreatic ductal adenocarcinoma. Septicaemia and pulmonary oedema were suspected to be the cause of death, possibly due to bacterial translocation through the small intestinal mucosa, for which peristalsis had most likely been hampered by SEP.

**Table 1:** Primary antibodies used for immunohistochemistry

Antibody to	Host	Type, clone	Dilution	Source	Catalogue number
CK^a^	Mouse	Monoclonal, AE1/AE3	1:500	Leica	NCL-L-AE1/AE3
Vimentin	Mouse	Monoclonal, V9	1:500	Leica	NCL-VIM-V9
CEA^b,e^	Mouse	Monoclonal, 12–140-10	1:500	Leica	NCL-CEA-2
WT1^c,e^	Mouse	Monoclonal, WT49	1:100	Leica	NCL-L-WT1–562
CGA^d^	Mouse	Monoclonal, 5H7	1:500	Leica	NCL-CHROM-430
Amylase	Sheep	Polyclonal	1:500	Biogenesis	0480–0404

^a^*CK* Cytokeratin

^b^*CEA* Carcinoembryonic Antigen

^c^*WT1* Wilms tumour 1

^d^*CGA* Chromogranin A

^e^Cross-reactivity to canine tissue was not guaranteed by the subcontractor for IHC

**Table 2:** Results of immunohistochemistry

Antibody	Tumor cells within pancreas	Tumor cells within peritoneum	Non neoplastic pancreatic duct epithelial cells
CK^a^	+	+	+
Vimentin	–	–	–
CEA^b,e^	–	–	–
WT1^c,e^	–	–	–
CGA^d^	–	–	–
Amylase	–	–	–

^a^*CK* Cytokeratin

^b^*CEA* Carcinoembryonic Antigen

^c^*WT1* Wilms tumour 1

^d^*CGA* Chromogranin A

^e^Cross-reactivity to canine tissue was not guaranteed by the subcontractor for IHC

## Discussion and conclusions

SEP is a rare condition in veterinary medicine that manifests as severe peritonitis with encapsulation of abdominal organs by thick collagenous connective tissue. Thirteen cases of SEP have been reported in dogs [1, 3–5, 10–15]. Common clinical symptoms of SEP in dogs include vomiting, anorexia, abdominal pain, and weight loss. In human cases of SEP, gastrointestinal obstruction is a common complication and thought to be a major cause of gastrointestinal clinical signs and abdominal pain [2]. The incidence of gastrointestinal obstruction in canine SEP is lower than that in humans, and only one case of pyloric obstruction secondary to SEP has been reported [13]. In the present case, no gastrointestinal obstruction was observed during the first visit. The initial clinical symptoms observed in the dog may have been associated with peritonitis.

Canine SEP is commonly diagnosed during exploratory laparotomy [1, 3–5]. Image analysis is useful as an adjunct for the diagnosis of SEP. Peritoneal effusion, thickened peritoneal line, and intestinal encapsulation are common CT findings in canine SEP [4, 11, 12]. In our case, peritoneal effusion with a thickened peritoneal line was found on CT at the first visit. Although encapsulation of the abdominal organs was not detected by either CT or laparoscopy at this time, the histopathological findings of the gastric serosal biopsy were consistent with those of SEP. Based on the imaging analysis, we speculated that the abdominal mass was the omentum covered by fibrous connective tissue. By the time of death of the dog, however, peritoneal sclerosis had advanced to cover the entire abdominal organs. Although the final diagnosis of SEP was made at the time of autopsy, the initial SEP lesion was successfully detected using CT, laparoscopy, and peritoneal biopsy.

Canine pancreatic exocrine tumours, which commonly originate from the acinar and rarely from duct epithelial cells, show aggressive behaviour with frequent metastasis [7]. Common clinical signs of pancreatic exocrine tumours include lethargy, anorexia, vomiting, and abdominal pain. In humans, contrary to canine epidemiology, ductal carcinoma is far more common than its exocrine counterparts in pancreatic cancers [16]. This tumour carries poor prognosis (5-year survival rate is approximately 5%) because of the absence of effective methods for early diagnosis and the aggressive nature of this tumour in humans [16]. According to the literature, the key pathologic features of well and moderately differentiated pancreatic ductal adenocarcinomas are as follows:1) glandular and ductal structures with a haphazard proliferating pattern, 2) marked desmoplasia, and 3) production of sialo-type and sulphated acid mucins [16]. The histopathological features of neoplastic epithelial cells in the present report are consistent with the characteristics of human pancreatic ductal adenocarcinoma. There is no IHC panel to establish a diagnosis of pancreatic ductal adenocarcinoma in human medicine. We understood this restriction and applied IHC to our case to rule out a differential diagnosis. The possibility of pancreatic acinar cell carcinoma was eliminated based on a negative reaction to anti-amylase antibody, as well as the absence of zymogen-like granules in the cytoplasm of the neoplastic cells [17]. Pancreatic neuroendocrine tumours were ruled out due to a negative reaction to chromogranin A and the lack of a typical proliferating pattern of neuroendocrine tumours [18]. Another important differential diagnosis in the present case was sclerosing peritoneal mesothelioma (SPM). Mesothelioma generally originates from the mesothelial lining of the thoracic, abdominal, and pericardial cavities and vaginal tunics of the scrotum and typically proliferates in epithelioid, sarcomatoid, or biphasic patterns [19]. SPM is a very rare variant of mesothelioma, characterised by abundant background fibrous tissue, despite few neoplastic mesothelial cells [19]. According to previous reports, canine SPM never proliferated in a tubular pattern, unlike in our case, but proliferated in nests, clusters, or individualised patterns [20, 21]. IHC results, especially the lack of vimentin or WT1 immunophenotype, also precluded the diagnosis of SPM in our case, although the antibody to WT1 was not reactive to canine tissues in our experimental setting. However, it has been demonstrated that the antibody to WT1 can be a marker of canine mesotheliomas; therefore, our IHC results would be regarded as legitimate [22].

In the present case, all three previous laparoscopic skin ports insertion sites showed abnormal postsurgical swelling, which was histologically demonstrated to be metastasis of pancreatic ductal adenocarcinoma (Figs. 6, 7). In humans, postsurgical port-site metastasis of malignant tumours has been reported to occur in 1 to 2% of patients [23], especially in the field of urology [24]. To the best of our knowledge, there are no such reports in the veterinary literature. Malignant tumours such as pancreatic ductal adenocarcinoma may be a potential cause of laparoscopic port-site metastasis. There is a need to carefully prevent this type of dissemination or metastasis of malignant tumours by regular and careful employment of laparoscopic specimen retrieval bags and/or by adequate procedures to protect the surface of laparoscopic ports when dealing with cases of potential malignant neoplasms. In this case, no abnormalities were found in the pancreas on the CT scan. However, since histologic changes suggestive of SEP were detected in the biopsy specimen taken at the first visit, it is likely that the pancreatic lesion was subtle enough to escape CT scrutinisation. Metastasis of tumour cells in the laparoscopic skin ports suggests the onset of pancreatic ductal adenocarcinoma at the time of laparoscopy and CT.

In this case, pancreatic ductal adenocarcinoma was observed concurrently with SEP. The previously reported underlying causes of SEP in dogs include foreign body ingestion (e.g. fibreglass and stick), steatitis, leishmaniasis, abnormal liver development, and neoplasia [1, 3–5, 10–15]. Only one case of hepatocellular carcinoma has been reported as a tumour-related SEP in dogs [11]. In humans, abdominal tumours such as pancreatic adenocarcinoma, midgut neuroendocrine tumours, and gastric carcinoma have been reported as underlying diseases of SEP [8, 9, 25, 26]. In human pancreatic tumours, rupture of the pancreatic ductal tissue causes leakage of pancreatic enzymes, leading to peritoneal inflammation with a desmoplastic stromal reaction [9]. In particular, human pancreatic adenocarcinoma of ductal origin causes prominent fibrosis in the pancreas, which is associated with the prognosis of this tumour [27]. In this case, marked desmoplasia was observed with extensive peritoneal dissemination of tumour cells, which is consistent with the above-mentioned features of human pancreatic ductal adenocarcinoma. We believe that this is the first case of canine SEP with pancreatic ductal adenocarcinoma to the best of our knowledge.

The prognosis of SEP in dogs depends on the underlying disease but is generally considered to be poor. Previous reports have shown that 93% of SEP cases die within 13 months [1, 3–5, 10–15]. To date, treatment of SEP in dogs has not been established. Only one report has demonstrated successful treatment of SEP in a dog with traumatic abdominal penetration injury using methylprednisolone and tamoxifen [4]. Tamoxifen has a supportive effect on metalloproteinase synthesis, promotes mesothelial healing, and assists in preventing the formation of new fibrous adhesions in the visceral peritoneum [28]. In this case, no response was observed to prednisolone and tamoxifen treatment, and the patient died 61 days after the first visit. The poor prognosis in this case was most likely associated with the concurrent pancreatic ductal adenocarcinoma. In a retrospective study of 23 dogs with pancreatic exocrine tumours, the median survival time was 1 d. In this report, a large number of dogs were euthanised immediately after diagnosis because distant metastases were observed at the time of diagnosis [7]. Another retrospective study of dogs reported that all cases of pancreatic tumours of canine ductal origin had metastasis in abdominal organs such as the liver, spleen, and intestine, and were euthanised at the time of diagnosis [6].

This report describes a case of SEP with pancreatic ductal adenocarcinoma in a dog. The dog had a poor prognosis, and a definitive diagnosis was confirmed by autopsy. As both SEP and pancreatic ductal tumours are extremely rare in dogs, further studies are warranted to clarify the clinicopathological features of both diseases.

## Data Availability

All data generated during this study are included in this article.

## Abbreviations

SEP: Sclerosing encapsulating peritonitis
CT: Computed tomography
PAS: Periodic acid-Schiff
IHC: Immunohistochemistry
CK: Cytokeratin
CEA: Carcinoembryonic Antigen
CGA: Chromogranin A
SPM: Sclerosing peritoneal mesothelioma
WT1: Wilms tumour 1
